# Phrenology

**Published:** 1851-07

**Authors:** 


*Phrenology.*—Many interesting facts are exhibited through the medium of occasional accidents which, if they do not go to establish general rules or support general laws, certainly justify, and should illicit the attention of such men as can unveil the obscurity by which they are surrounded, and throw light upon matters of much interest; such is the condition of phrenology, struggling by the assistance of many isolated facts, it still remains unestablished in the scientific world as one of its supports, or as a child of its nurture and care. That there is found much undue hostility towards any new science or philosophy is too true, yet the researches of all science, having nature for its subject, and truth its object, must eventuate, in the undisputed exemplification of the want or existence of these great principles. We should think that Magendie's and Bouillard's system of investigation is incomplete and should be abandoned, as also that of Sir Edward Hume's and others, from the fact that mutilation of any part of the living brain destroys or involves the functions of the nervous system generally, and functional disarrangements often ensue without visible organic lesion, and are symptomatic of diseases in other organs. Dr. Gall's method is greatly approved by those who have given much attention to the matter, and perhaps possesses many advantages. With these few remarks, we give a report of a case found in the London Medical Lancet, of much interest, and is as follows:

By C. W. Latham, Esq., M. R. C .S., London, Eng. A circumstance in the history of a case of congestive apoplexy, which has recently been under my care, the subject of which is now convalescent, appears to me sufficiently curious to interest some of my professional brethren, especially such as advocate the doctrines of phrenology.

"James T——, aged sixty-three, an old seaman, but who has been employed as a brewer's servant for the last thirty years, about two years ago, while in the employ of Messrs. Truman, Hanbury and Co., received a severe blow on the top of his head, from the falling of a heavy cellar-flap, on the under surface of which were affixed two iron bolts; one of these struck him on the right parietal bone, at its posterior superior angle, close by the posterior fontanelle. He was for a short time rendered insensible by the blow, but quickly recovered and returned to his work. Before this accident he was fond of music, could (to use his own expression) 'sing a very good song for a commoner,' was in the habit of whistling and singing at his work, and possessed a tolerable facility in acquiring new airs. From that time, however, he lost all musical power; on attempting to sing the most familiar air, he could not get over more than a single bar, when he became confused, and struck out into two or three other different tunes. He recognised any music which he had previously heard, but was quite incapable of singing it himself, or of acquiring anything new, as before.

"This is the only injury or inconvenience which he sustained; his memory, in other respects, and all his other faculties, are unimpaired; but although he has repeatedly put himself to the test since, he finds that his musical powers are still thus in abeyance."

